# Specimen records of spiders (Arachnida: Araneae) by monthly census for 3 years in forest areas of Yakushima Island, Japan

**DOI:** 10.3897/BDJ.5.e14789

**Published:** 2017-07-25

**Authors:** Takeshi Osawa, Yuki G Baba, Tatsumi Suguro, Noriaki Naya, Takeo Yamauchi

**Affiliations:** 1 Institute for Agro-environmental Sciences, Tsukuba, Japan; 2 Keio Yochisha Elementary School, Tokyo, Japan; 3 Graduate School of Life and Environmental Sciences, University of Tsukuba, Tsukuba, Japan; 4 Museum of Nature and Human Activities, Hyogo, Sanda, Japan

**Keywords:** Araneae, Darwin Core Archive, GBIF, monitoring, sample event data

## Abstract

**Background:**

Spiders (Arachnida: Araneae) are a classic indicator taxon for evaluating the health of natural environments. However, studies of spiders’ responses to forest succession under natural and anthropogenic disturbance regimes are lacking. Yakushima Island in southwestern Japan has a unique forest ecosystem, and part of the island is designated as a world natural heritage site by UNESCO. Approximately 90% of Yakushima is covered by forest, including both plantations and natural forests.

**New information:**

We made an inventory of spiders on Yakushima Island by collecting specimens in five forests (two plantations and three natural forests) with Malaise and window traps from 2006 to 2008 (a total of 637 traps). We collected 3487 specimens, representing 31 families and 165 species or morphotypes, including undescribed and unidentified species. All specimens were preserved in 70% ethanol, and all data were gathered into a Darwin Core Archives as sample event data. The data set is available from the GBIF network (http://www.gbif.org/dataset/f851fd75-32b2-4a23-8046-9c8ae7013a3c). Because there have been no spider inventories based on such a systematic trapping survey in Japan, this data set provides new insight into the biodiversity on Yakushima Island.

## Introduction

Taxa that are suitable as ecological indicators are sensitive to environmental changes and therefore demonstrate negative effects of anthropogenic disturbance on the processes or functioning of an ecosystem (Pearce and Venier 2006). Although several animal taxa have been proposed as environmental indicators, both ground beetles and spiders are widely used as indicator taxa (Bedford and Usher 1994, Pearce and Venier 2006, Buchholz 2010, Tanaka and Ihara 2012, Osawa et al. 2014). Both taxa are economically and logistically feasible to survey, being sampled simultaneously in large numbers using passive sampling techniques (Thiele 1977, Wise 1995, Pearce and Venier 2006). Although many studies have used these taxa as indicators, some knowledge gaps remain. In particular, Peace and Venier (2006) advocated using these taxa as indicators in studies of responses to forest succession under natural and anthropogenic disturbance regimes in order to provide forest managers with better decision tools. Thus, establishing an inventory of these organisms in several types of forests is important for developing conservation and environmental management strategies.

Yakushima Island in southwestern Japan is home to a unique forest ecosystem in which a gradient of subtropical to subarctic vegetation remains along the elevational gradient (Agetsuma et al. 1994). Part of the island has been designated as a world natural heritage site by UNESCO because of the unique fauna, flora, and landscape (http://whc.unesco.org/en/list/662 accessed 14, July, 2017). Many endemic species are found on Yakushima Island, but we still lack basic information on the island’s fauna (Watanabe and T. Yamauchi 2014). About 90% of Yakushima Island’s 505 km^2^ area is covered by forest, including natural rain forest and cedar plantations Tokumaru (2003). In addition to UNESCO, both the Japanese government and the local Kagoshima government are involved in nature conservation efforts on Yakushima Island (Okano and Matsuda 2013, Tokumaru 2003), but these efforts require more knowledge, particularly regarding environmental indicator species.

We surveyed spider (Araneae) species from 2006 to 2008 to make an inventory of spiders across different types of forests on Yakushima Island. We conducted monthly censuses with two types of trap in both plantations and natural forests. The aim of this data paper is to share the inventory of spider species on Yakushima Island using the standard Darwin Core data format to increase its availability. This is the first inventory of spiders in Japan constructed by using a systematic sampling method and will provide important knowledge on the biodiversity of Yakushima Island.

## Project description

### Title

Sustainability and biodiversity assessment on forest utilization options

### Study area description

**Figure 1.**
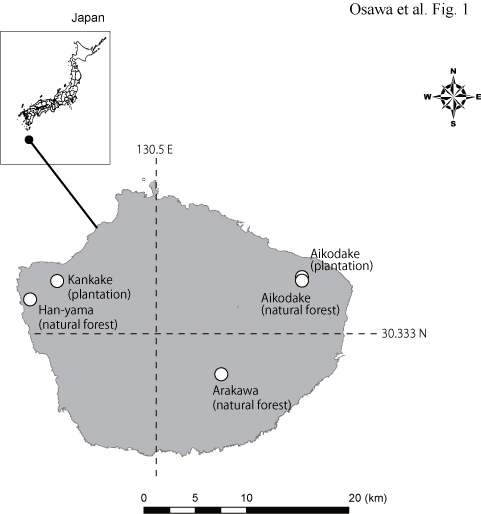
Study area and monitoring sites.

Yakushima Island is a granite island (ca. 505 km^2^) surrounded by sedimentary rocks and is located approximately 70 km south of Kyushu, Japan (Fig. 1). The island has precipitous terrain, including mountains approximately 2000 m in elevation, and about 90% of the island is covered by forest. On the plains, the average annual temperature is 19.1°C and the average annual precipitation exceeds 4000 mm, with 10,000 mm of precipitation in the mountainous areas. In 1993, UNESCO designated 21% of Yakushima as a world natural heritage site because of the unique fauna, flora, and landscape (http://whc.unesco.org/en/list/662 accessed at 15, July, 2017).

## Sampling methods

### Study extent

Fig. 1

### Sampling description

Insect traps were set at five sites in three regions: old-growth evergreen forests (Aikodake and Han-yama) and neighboring 40-year-old Japanese cedar (*Cryptomeria japonica*) plantation forests (Aikodake and Kankake) in the low mountainous region (150-250 m elevation), and an old-growth mixed forest (Arakawa) higher in the mountains (1200 m elevation) (Fig. 1). The old-growth evergreen forest and the Japanese cedar plantation in Aikodake are part of a continuous forest, and they are only 100 m away from each other.

A Townes-type Malaise trap and IBOY-type window trap were used for sampling spiders. These types of trap are suited to a monitoring program in forest habitat because they capture a wide range of spiders, excluding some ground-dwelling forms. The Malaise trap had openings with a height of 1.8 m and a length of 1.8 m on both sides, and the attached bottles contain a mixture of 70% ethanol and a small amount of ethylene glycol for fixation and preservation of samples. Three Malaise traps were set at each study site at intervals of about 20 m (15 in total). Malaise traps were set continuously from July 2006 to March 2008, and samples were collected about once every month. The IBOY window trap consists of crossed transparent acrylic collision boards on a yellow bucket with a diameter of 36 cm, in which 1.5 L of water containing 10 ml of neutral detergent and 10% acetic acid aqueous solution is placed. We hunged these window traps at a height of about 30 cm above the ground. The window traps were set for 3 days in the latter half of each month from July 2006 to February 2008. The samples collected by each trap were brought back to the laboratory, and spiders were identified and counted.

## Geographic coverage

### Description

The census was conducted in three regions on Yakushima: Aikodake, with both natural (30.381°N, 130.627°E) and cedar plantation forest (30.384°N, 130.627°E); Kankake, with Japanese cedar plantation forest (30.381°N, 130.412°E); Han-yama, with natural forest (30.364°N, 130.389°E); and Arakawa, with natural forest (30.299°N, 130.556°E) (Fig. 1).

## Taxonomic coverage

### Description

In total, 3487 individuals belonging to 162 species, including morphotypes from 31 families, were collected during the censuses. Of these collections, two records have already been published as papers (Baba et al. 2016, Baba et al. 2015).

All specimens were identified by the authors according to Ono (2009) and scientific names were determined according to the World Spider Catalog Natural History Museum (2017). If we could not obtain sufficient information for proper identification, we determined the order, family, and genus of each species on the basis of the relevant morphotypes. Thus, some records have only order, family, or genus rank for the taxonomic information. The species name genus and family sequence are arranged in alphabetical order.

Order: Araneae

Family: Agelenidae, Anyphaenidae, Araneidae, Atypidae, Clubionidae, Ctenidae, Ctenizidae, Dictynidae, Eutichuridae, Gnaphosidae, Leptonetidae, Linyphiidae, Liphistiidae, Lycosidae, Miturgidae, Mysmenidae, Oonopidae, Oxyopidae, Philodromidae, Phrurolithidae, Pimoidae, Pisauridae, Salticidae, Segestriidae, Sparassidae, Tetragnathidae, Theridiidae, Theridiosomatidae, Thomisidae, Zodariidae.

## Temporal coverage

### Notes

The term of this census was 3 years (2006–2008). The 2006 census was from July to December, with 227 events (traps) in total; in 2007 from January to December, with 351 events in total; and in 2008 from January to March, with 58 events in total. Each event has a term of 3 days to about 1 month.

## Usage rights

### Use license

Other

### IP rights notes

Creative Commons CC-BY 4.0

## Data resources

### Data package title

dwca-yakushimaspyder01-v1.1

### Resource link

http://www.gbif.org/dataset/f851fd75-32b2-4a23-8046-9c8ae7013a3c

### Number of data sets

1

### Data set 1.

#### Data set name

Specimen records of spiders (Arachnida: Araneae) by monthly census for 3 years in forest areas of Yakushima Island, Japan

#### Data format

Darwin Core Archive

#### Number of columns

1

#### Download URL

http://osawa.nomaki.jp/dl/dwca-yakushimaspyder01-v1.1.zip http://www.gbif.org/dataset/f851fd75-32b2-4a23-8046-9c8ae7013a3c

#### Description

The data sets are stored on the website of the corresponding author and have been uploaded to the JBIF (GBIF Japan) portal.

**Data set 1.** 

Column label	Column description
darwin core event	darwin core event

## References

[B3665682] Agetsuma Naoki, Sugiura Hideki, Tanaka Toshiaki (1994). Influences of Seibu Road on the World Heritage Forest of Yakushima Island, Japan. Primate Research.

[B3681473] Baba Yuki G., Suguro Tatsumi, Naya Noriaki, Yamauchi Takeo (2016). A gynandromorph of the funnel-web spider *Allagelena
opulenta* (Araneae: Agelenidae). Acta Arachnologica.

[B3681483] Baba Y. G., Suguro T., Yamauchi T. (2015). A record of *Anepsion
japonicum* (Araneae: Araneidae) from Yakushima Island, Ryukyu Islands, Japan, after an interval of 47 years. Fauna Ryukyuana.

[B3665692] Bedford S. E., Usher M. B. (1994). Distribution of arthropod species across the margins of farm woodlands. Agriculture, Ecosystems & Environment.

[B3665702] Buchholz Sascha (2010). Ground spider assemblages as indicators for habitat structure in inland sand ecosystems. Biodiversity and Conservation.

[B3665814] Museum Natural History (2017). World Spider Catalog. Version 18.0.

[B3665732] Okano Takahiro, Matsuda Hiroyuki (2013). Biocultural diversity of Yakushima Island: Mountains, beaches, and sea. Journal of Marine and Island Cultures.

[B3665785] Ono H. (2009). The Spiders of Japan: with keys to the families and genera and illustrations of the species.

[B3665742] Osawa Takeshi, Watanabe Kyohei, Ikeda Hiroaki, Yamamoto Shori (2014). New approach for evaluating habitat stability using scarce records for both historical and contemporary specimens: a case study using Carabidae specimen records. Entomological Science.

[B3665752] Pearce Jennie L., Venier Lisa A. (2006). The use of ground beetles (Coleoptera: Carabidae) and spiders (Araneae) as bioindicators of sustainable forest management: A review. Ecological Indicators.

[B3665762] Tanaka Koichi, Ihara Fumio (2012). Biodiversity Research for the Development of Indicator Organisms in Environment-Preserving Agriculture. Ecological Research Monographs.

[B3665837] Thiele H. U. (1977). Carabid beetles in their environments: a study on habitat selection by adaptation in physiology and behavior. Zoophysiology and Ecology 10.

[B3665794] Tokumaru H. (2003). Nature conservation on Yakushima Island: Kagoshima Prefecture’s efforts. Global Environmental Research English Edition.

[B3665804] Watanabe K., T. Yamauchi (2014). Records of ichneumonid wasps (Hymenoptera) from Yakushima Island, the Ryukyu Islands, Japan. Japanese Journal of Systematic Entomology.

[B3665851] Wise D. H. (1995). Spiders in ecological webs.

